# A report on DNA sequence determinants in gene expression

**DOI:** 10.6026/97320630016422

**Published:** 2020-05-31

**Authors:** Ravail Singh, Yengkhom Sophiarani

**Affiliations:** 1 Indian Institute of Integrative Medicine, CSIR, Canal Road, Jammu-180001; 2 Department of Biotechnology, Assam University, Silchar-788011, Assam, India

**Keywords:** Gene expression, codon, amino acid, genome

## Abstract

The biased usage of nucleotides in coding sequence and its correlation with gene expression has been observed in several studies. A complex set of interactions between genes and other components of the expression system determine the amount of proteins produced from coding sequences. It is known that the elongation rate of polypeptide chain is affected by both codon usage bias and specific amino acid compositional constraints. Therefore, it is of interest to review local DNA-sequence elements and other positional as well as combinatorial constraints that play significant role in gene expression.

## Background

DNA is the building block of life. The DNA sequence of genes and genomes is the blueprint of the gene function. All the information related to hereditary and species evolution is contained in these macromolecules. DNA sequences form the basis for all bioinformatic analysis tools and resources. In bioinformatics, these sequences are analyzed using a wide range of analytical methods to discover genes, its features, function, structure, or evolution [1]. The discovery of DNA sequencing technology has made available many sequence datasets from different genomes over the last three decades. Subsequently, it has become apparent that the nucleotide composition varies from genome to genome and is one of the major perplexing characteristics of each genome. DNA sequence analysis is widely used as a highly accurate and flexible tool for classifying and identifying organisms [2]. All the nucleotide sequences present in a genome do not code for proteins. Protein coding sequences usually start with an initiator codon (usually AUG) and end with a terminator codon (either UAA or UGA or UAG) in standard genetic code [2]. Any continuous stretch of DNA that has the tendency to encode a specific protein is firstly transcribed as a messenger RNA then translated into a protein. The protein translation machinery present within any organism reads the DNA sequence in groups of three nucleotides (codons), which eventually determine the outline of amino acids that will appear in the ultimate protein. Therefore, a single-stranded DNA and a double-stranded DNA have three and six distinct reading frames, respectively. An open reading frame (ORF) is a DNA sequence that has the potential to code for a protein. Out of the six ORFs, only one is used in translating a gene (in eukaryotes), and this is often the longest ORF. The nucleotide sequence present within each ORF provides the instruction for encoding different proteins. A specific region of the DNA sequence is destined to code a protein for a specific function. For example, variation in the flower color among different plants belonging to the very subfamily is due to the variation in a specific region of a gene, which determines the flower color. These variations of a particular gene are called alleles, which produce the alternative or different forms of one trait. Gene expression is a fundamental cellular process through which proteins are synthesized within all living cells using the nature gifted genetic codes. Gene expression is a complex biological process separated into several phages, including synthesis and processing of mRNA, export (in eukaryotes), translation, and decay regulated at a variety of steps, from DNA to RNA to protein. Four major steps are involved in regulating the rate of gene expression, (a) timing and rate of transcription initiation and elongation; (b) processing of transcripts; (c) rate of transcript degradation, and (d) post-transcriptional modification of transcripts.

**Figure 1:**
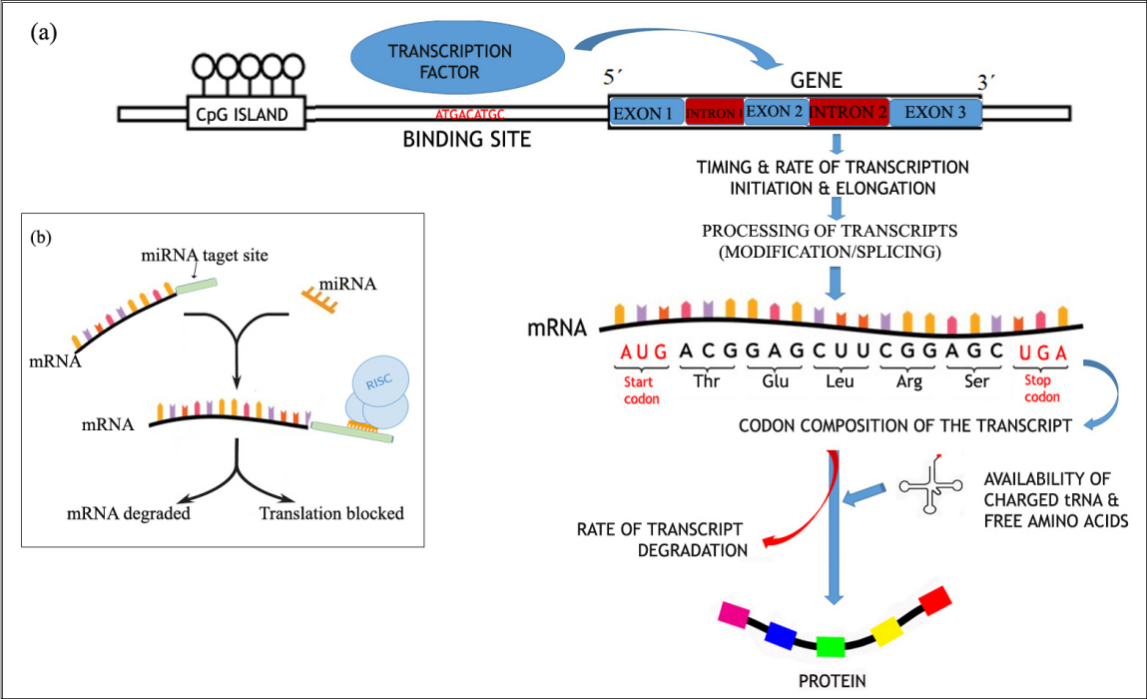
:(a) Role of various factors that operate at the various stages of gene expression, (b) MicroRNA mediated gene silencing

The physiological state of a cell is determined by the absolute concentration of proteins. Maintaining the amount of proteins at a steady state is a crucial feature of controlling gene expression. The process of gene expression is far more complex in eukaryotes than prokaryotes and the concentration at which a particular protein is produced depends on various factors [3]. The unique DNA sequence assets present in each gene can have conspicuous effects on its expression. Various sequence determinants present within a gene and their role in expression (tissue specific), operate through complex pathways, and determine the differential regulation of gene expression during development and differentiation. Advances in computational biology and its genetic engineering applications have opened the doors for a systematic study of the compositional constraints that affect gene expression (Figure 1). We here discuss the sequence determinants with some astonishing results emerged from extensive research done in the field of gene expression and genetic engineering.

## Factors affecting gene expression level:

CpG Islands and transcription factors:

CpG islands are typically several hundred bases to several kilo bases long interspersed DNA sequences, rich in GC-rich, and predominantly non-methylated [4]. CpG islands play a significant role in the regulation of genes by epigenetic changes [5]. In situ hybridization on meta-phase chromosomes from blood lymphocytes revealed that the GC-rich genes localized in the GC-rich isochores express at a rate higher than others [6]. The lengths of CpG islands vary and show relevant variation in their functions [7]. CpG islands commonly found in the region where vertebrate genes begin transcription, perhaps in the case of all housekeeping genes and the genes expressed frequently in a cell. Approximately 70% promoters identified from the vertebrate genome were found to be associated with the CpG islands. CpG islands located over the transcription start point are known as start CpGs, whereas other islands are known as non-start CpGs. The promoter-associated CpG islands are structurally different from others [8]. CpG island regulates gene expression by influencing the local chromatin structure [4], often lacks TATA boxes and displays heterogeneous transcriptional start sites enriched with transcription factor-binding motifs (E2F, Sp1, ETS, and Nrf-1) [9]. Methylation or demethylation of CpG islands results in repression or activation of gene expression, respectively. Altered CpG island methylation regulates gene expression by influencing the physical access of transcriptional factors at CpG islands [10]. Several reports on CpG methylation showed that altered methylation at CpG attract several other methyl-CpG-binding-domain, which eventually recruits histone deacetylases (HDAC) and stops the transcription processes [11]. Previous reports showed that the regulatory CpGs (usually at the promoter region) on several protooncogenes/oncogenes are inappropriately methylated [12]. Antigens of the cancer-testis (CT) shows difference in methylation between normal and cancer cells, the former one has methylated CpGs at the CT antigens promoter region [13]. Long CpG island promoters are generally found in a few tissues. Furthermore, the genes associated with promoter CpG islands were more frequently expressed as compared to the genes without CpG islands [7]. Transcriptional factors and co-factors can vary for each gene. The difference in the sequence length of the island and their varied role in gene expression are thought to be due to the accessibility of the larger region for added transcriptional factors.

Transcription factors (TFs), control gene expression by binding to regulatory sequences, such as enhancer sequences, recruit co-activators and RNA polymerase to target genes. The biophysical interaction between DNA and protein structure determines the ability of the TFs to bind to a regulatory sequence [14]. TFs recognize unique cis-regulatory regions of the core promoter elements, which recruit the transcription machinery [15]. Many TFs are involved in the transcription of different promoters while some are very selective to a few promoters [16]. Their influence can be positive or negative, depends largely on the presence of several other functional domains and the overall impact of the entire TF complex. Differences in the concentration of TFs and co-factors influence the timing as well as the rate of transcription, thereby the expression of a gene [17][18]. For the mitochondrial genes of vertebrates the transcription factor A (mtTFA) plays the key role for the regulation of gene expression [19]. In the year 2000, Batlle et al., identified that a transcription factor (snail protein) plays a crucial role in epithelial tumour progression by down-regulating the adhesion protein E-cadherin [20]. They observed that the inhibition of this TF restores the expression of the E-cadherin gene. GATA-3 is essential for Th2 cytokine gene expression in CD4 T cells, a major target for modifying the immune responses in many immunological conditions [21]. Under dehydration and low temperature, dehydration-responsive element binding protein 1 and 2 acts as trans-acting factors in Arabidopsis [22]. Another TF named NF-κB was found to be involved in human diseases gets activated by oncoproteins to induce cellular transformation. It has been reported that HIV and many other viruses induce NF-κB activation, because of its ability to regulate cell cycle, DNA replication, and apoptosis [23]. The transcription factor NKX2-5 is needed for the maturation and maintenance of atrioventricular node function and any mutation in this TF encoding gene leads to congenital heart disease [24]. Therefore, the development of ligand binding approaches to address these transcription factors may provide a new biomedical avenue to treat or prevent diseases.

## Variation in G+C content:

Genomic GC content varies significantly from small single cellular prokaryotes to multicellular eukaryotes due to two major factors: natural selection and mutation pressure [25]. Advancement in the field of sequencing technology and sequence-based analysis makes it possible to identify and compare genomes based on their sequence characteristics. The key cause of heterogeneity in GC content (location-specific) in prokaryotes is due to the presence of differentially expressed genes with varying level of nucleotide bias [26]. GC content variation between different genomic regions of the eukaryotes is an adaptation to higher body temperature, and it plays a fundamental role in genome organization. Some genomic regions of the eukaryotes are found to be very GC rich as well as gene rich, whereas some other regions are GC poor [27]. This variation in the nucleotide composition in different genomic region makes use of the codons with varying composition in different genes present at those particular genomic positions [28]. Furthermore, several studies have demonstrated that GC content correlates with the gene expression level [29]. Newman et al., (2016) disclosed that the increased production of their studied proteins was not due to increased translation but for the change in transcription of GC-optimized codons [30]. This suggests that evolutionary force is responsible for maintaining the conserved genomic regions and for the GC rich codon usage of genes in eukaryotes. Guo et al. 2007, reported that the cereal crops, particularly maize provide a plausible evolutionary mechanism for genes with a strong GC bias [31]. The telomeric region shows an unusual pattern of GC substitution different from the rest of the genome. In addition to the role on gene expression level the genomic GC content influences many other important genomic features: (i) gene density; (ii) recombination rate; (iii) distribution of repetitive elements (SINE and LINE); (iv) replication timing; (v) methylation pattern, and (vi) distribution of the transposable elements [32].

## Codon usage bias:

Codon usage bias (CUB) is the phenomenon where some codons encoding a specific amino acid are used more preferentially over others. It is a complex process influenced by several factors like GC content, protein abundance, mRNA folding etc. A balance between mutation, selection, and genetic drift ensures the strength of CUB [33]. Molecular evolutionary investigations suggest that codon usage represents a characteristic pattern of preference in each organism, and it also differs within in a single genome at different locations [34]. The codon bias is the most prominent among highly expressed genes. The influence of codon bias on gene expression is a topic of active debate. Codon bias, first studied on the model organism Escherichia coli (E. coli), revealed strong bias in codon usage for genes encoding abundant proteins. CUB causes variation in GC content, particularly at the synonymous third codon position. CUB has been studied extensively in the past two decades but the relative contribution of the two major determinants of codon usage bias i.e., mutation and selection is still mysterious. Genetic engineering approach utilizes the information generated from CUB analysis to increase protein production. Research on CUB has shown its role in regulating protein expression by affecting elongation speed. However, recent studies have shown that translation efficiency is mainly affected by the efficiency of the initiation of translation, where CUB plays a minor role [35]. The approach of codon optimization used in the process of genetic engineering affects protein conformation, and function, and also increases immunogenicity, but reduces efficacy [36]. Heterologous protein expression for increased protein production is a multidimensional optimization problem [37]. Therefore, while optimizing codons in a gene, other factors like mRNA folding, bendability and stability should be taken into consideration, particularly for in vivo applications [38]. Furthermore, it was observed that the first 30-40 nucleotides of a gene show a different pattern of codon usage from the rest of the gene sequence [39]. Further, the anterior region of coding sequence undergoes selection pressure and regulates gene expression through mRNA folding during translation initiation [40]. Previous studies suggest that synonymous sites are functionally neutral, but some recent findings contradict it, i.e. synonymous mutations are associated with diseases [41]. Mendelian disease-causing synonymous SNPs, for example, has a comparable effect size as non-synonymous SNPs in association studies of human disease [42]. In addition, substantial associations have been identified in the case of Alzheimer's disease between genetic variants containing a favored codon in minor alleles and a rare codon in the major allele [43]. These results provide an insight into the roles of CUB in forming different protein structures and can be used in rare variant association research to boost detection efficiency.

## mRNA folding energy:

The folding energy of an mRNA depends on the coding sequence of a gene. With the high folding score, mRNAs fold more strongly. Intra-molecular hydrogen bonds and base stacking interactions between nucleotide pairs determine the secondary structure of the mRNA molecule. Furthermore, the function of an mRNA closely resembles its structure. The secondary structure of mRNA can be predicted with the aid of bioinformatics, since mRNA molecules are more conserved in secondary structure than their primary base sequences [44]. Enzymatic methods can also be used for structural inferences [45]. A given mRNA may have a different structural conformations at different locations depending on the sequence properties. Furthermore, the mRNA folding pattern is environment-dependent [46]. Therefore, during the rapid cell growth a single gene might be folded somewhat differently to allow faster translation and elongation than steady state condition. The correlation between mRNA folding and translation is complicated and has opposite impacts [47]. Highly expressed mRNAs’ folding structures undergo strong selection pressure to reduce ribosome sequestration for accelerated elongation. Researchers proposed that the folding of an mRNA influences ribosome binding and hence plays a crucial role in determining the gene expression level [48]. Selection pressure acts near the initiator region to decrease the folding energy, slow down the ribosomes and decrease translational efficiency. Kudla et al. 2009 analyzed the green fluorescent protein (GFP) from E. coli genome [49]. For the same gene they designed different coding sequences without altering the native amino acid composition. They observed that the folding energy of the initial 40 nucleotides from each mRNA showed strong correlation with the protein abundance values. Tuller et al., 2010 found a clear link between the genomic profile of the folding energy and the ribosome density demonstrated by the study of S. cerevisiae and E. coli transcriptomes [48]. This relationship means that highly structured mRNA holds back the velocity of ribosomal motion on mRNA, as the density of ribosomes is higher for lower ribosomal velocity assumed by constant ribosomal flux [50]. Genes having the tendency to express at high rate undergo strong selection pressure for stronger folding which results in the slow evolution of these genes [45]. These results altogether suggest that folding energy influences the global translation efficiency (translational initiation plus translation elongation). An error in protein folding and the accumulation of these misfolded proteins leads to amyloid diseases.

## Gene length:

The length of genes varies from gene to gene relating to their proper folding and function. The average gene length is usually longer in eukaryotes than prokaryotes. Gene length is a dynamic property. Length of the gene increases during the evolutionary process due to transposable elements. Therefore, the increased length of the eukaryotic genes indicates the evolutionary complexity associated with gene length [51]. Eukaryotic genes are rich in introns. Introns are the sites where transposable elements are introduced and also provide a mechanism for generating transcriptional complexity within the multicellular genomes via the preferable mixture of exons. Researchers found that the genes expressed at high level are shorter in size and tend to have shorter introns [52]. Large scale analysis on prokaryotic genes proved the relationship between gene length and expression level. Long gene requires substantial time to be expressed following its activation, for example, the largest known gene human dystrophin (primary transcript of 2.3Mb) requires approximately 16h for transcription. Although the gene dystrophin has a longer gene length required for encoding the proper number of amino acid to make it fully functional, it seems to be under selection pressure since the gene dystrophin is very long but has fewer introns [53]. Gene lengths show significant correlations with both gene duplication (negative) and alternative splicing (positive) [52]. Grishkevich and Yanai (2014) have shown that the relationship between gene duplication and alternative splicing is regulated by two key genetic properties, i.e. gene length and level of expression [52]. In the cortical neurons, longer genes associated with neuronal development and synapses were down-regulated by topoisomerase inhibitors via impairing transcription elongation [54], might impair neuronal function and lead to neurological disorder [55]. In addition, recent studies showed gene-length mediated shift in the expression of Rett syndrome (RTT) neurodevelopmental disorder [56]. Transcriptional timing is inherently influenced by gene length, provides a mechanism for temporal regulation of gene expression [57]. A research on Drosophila has shown that the gene length mediates developmental timing of gene expression [58]. These findings altogether suggest that gene length is an important factor influencing virtually all aspects of molecular evolution.

## Amino Acid Composition:

Proteins vary in their amino acid compositions, which depends largely on the locations where the proteins are destined to function. It is anticipated that natural selection could play a role in preserving or enhancing the protein activity, specificity or stability by favoring specific codons encoding corresponding amino acids at critical positions in the protein's primary structure. But in less constrained positions of the protein, a combination of both mutation pressure and genetic drift might act on the coding sequences to encode the specific amino acids in the protein [59]. Metabolic constraints on protein crystal structure include biosynthesis cost of amino acids, complexity of synthetic pathways, nutrients, protein synthesis accuracy and speed [59]. The correlations between the composition of amino acids and protein function are well documented for three lineages of life: prokaryotes, archaea and eukaryotes [60]. However, less attention was given to the relationship between the efficiency of protein biosynthesis and its primary structure. The degree to which the composition of amino acids is skewed to minimize metabolic costs ought to be a good measure of the amount of proteins synthesized from each gene per generation. Akashi et al., 2002 demonstrated that the frequency of certain amino acids varies in wide functional protein categories as a result of translation rate estimation [59]. The cost of amino acid biosynthesis ranges from 11.7 PO4 for less complex amino acids (eg: glycine and proline) to 74 PO4 for extremely complex amino acids (eg: tryptophan). The use of these less costly amino acids in highly expressed genes has an energetic benefit that can surpass 0.025% of the total energy expenditure. Costly amino acids namely Tryptophan, Phenylalanine, Histidine, Cysteine, and Leucine are found in less frequency in highly expressed genes, whereas the frequency of the less costly amino acids such as Glutamine, Asparagine, and Glycine are usually more in highly expressed genes. These results suggest the effect of natural selection to enhance metabolic efficiency by increasing the use of more costly amino acids in lowly expressed genes but the use of less costly amino acid in highly expressed genes, respectively. Moreover, a significant number of genes across all living species encodes proteins with amino acid repeats of different length and composition that play important role in overall protein structure and function [61]. In addition to its role as a substrate for protein synthesis, recently it was reviewed that amino acids in concert with hormones modulate various signal transduction pathways, which regulate mRNA translation [62]. The utilization of amino acids and its demand varies between healthy and disease conditions [63]. Any abnormality in the metabolic pathways of a specific amino acid leads to the accumulation of that amino acid, can evoke a toxicity syndrome which usually extends to central nervous syndrome (e.g. hypoglycemia) [64].

## tRNA abundance:

A tRNA (transfer ribonucleic acid) molecule typically contains 76 to 90 nucleotides that help decipher a mRNA sequence into a protein. Each tRNA recognizes specific amino acid and carries only one amino acid attached to its end at a time. When a tRNA binds to ribosome with its matching codon, it transfers a corresponding amino acid to the expanding polypeptide chain. The translation rate or the decoding rate of a codon depends on the speed of delivery of its translationally competent tRNA to the ribosome [65]. We all know about the redundancy of the genetic code i.e., 61 codons code for 20 amino acids. Hence, there must be an equal number of tRNA molecules for each of these codons but there is a large variation in the tRNA gene copy number per cell by up to 10 fold [66]. Soon after the experimental documentation of the correlation between the codon usage bias and tRNA abundance researchers tried to find out the relationship between the codon adaptation and the gene expression level [67]. Extensive research on gene expression found strong correlation with codon adaptation. Highly expressed genes that are enriched with optimal codons are recognized by abundant tRNAs and translated faster than codons read by low-abundance tRNAs [40]. The initial region at the 5' end of a gene has somewhat different nucleotide composition pattern generally recognized by tRNA species with lower intracellular abundance and provides several physiological benefits [68]. Individual tRNA expression varies in different tissues, and tRNAs decoding amino acids with specific chemical properties showed structured expression in multiple tissue types. Tissue-specific expressed gene and coordinated expression of tRNAs implicate its function in controlling translation and probably secondary processes in mammals [69]. Goodarzi et al., (2016) showed that specific tRNAs are up-regulated in human breast cancer cells as they gain metastatic activity [70]. Gorochowski et al., 2015 analyzed the role of tRNA abundance in mRNA folding and translation elongation [71]. They observed that the gene regions enriched with codons having more abundant tRNA has the propensity to form strong secondary structure. This structure eventually influences the translation elongation dynamics and enhances protein translation and leads to increased protein yield. Mutation in tRNA genes and its processing enzymes leads to a variety of complicated clinical phenotypes, for example, mutation in mitochondrial tRNA (mt-tRNA) causes mitochondrial myopathies [72].

## Presence of the correct 5'- cap and poly (A) tail in 3'-end region and the role of miRNA:

Soon after the post-transcriptional modification, all mRNA (except the replication dependent histone transcript) molecules in eukaryotes acquire a 5'- cap (m7GpppN) and a poly (A) tail at their 3'-ends. The 5'-cap structure in eukaryotes regulates the overall quality of mRNA products by inducing the translation activation frequency [73]. The mRNA poly (A) tail at the 3'-end region has a profound effect on mRNA bendability, translation rate, cell viability, growth, and development [74]. During the process of translation, synergistic effect of the poly (A) tail and the 5'- cap of the mRNA direct the ribosome to bind to the initiator region. The 5' m7G cap of eukaryotic mRNA recruits cellular proteins and mediates cap-related biological functions. Decades of research have established the importance of a proper cap structure for the optimal translation of functional messenger RNA [75]. It is an important regulation point of gene expression that protects mRNA from degradation, promotes transcription and nuclear export [76]. Researchers working in a cell free translation system revealed that poly (A) tail independently promotes the binding of the small ribosomal subunit [77]. Thus, poly (A) tails are also known as translational enhancer. Polyadenylation signals (PAS) are often considered as a distinguishing characteristic of eukaryotic genes. A highly conserved motif AAUAAA is present in almost all eukaryotic polyadenylated mRNAs found 10 to 30 nucleotides upstream of the cleavage site, essential for both cleavage and poly (A) addition as well as for promoting downstream transcriptional termination [78]. Approximately 30 to 40 nt downstream of the AAUAAA motif there is another additional region (less conserved, either U or GU rich or both) and the distance between the AAUAAA motif and these additional motifs determines the cleavage site i.e. site of poly (A) addition. Preiss and Hentze, 1998, showed that independently both the 5'-cap and the 3'-tail can promote translation but not enough to promote efficient translation [79]. These results suggested the need of proper cap and tail (closed –loop model) for efficient translation of an mRNA molecule.

MicroRNAs are short non-coding RNA molecules, which regulate gene expression at the transcription and post transcription level, generally bind to their target mRNAs 3 prime untranslated region. Recently, the structure and functions of this essential intracellular genomic regulator have been highlighted. MicroRNA binds with mRNA and inhibits protein translation or destabilizes target transcript. The length of the 3' UTRs decides the density of miRNAs binding to the mRNAs [80]. Extensive research on miRNA revealed that the seed region is responsible for target recognition, which pairs fully with the target region [81]. They play crucial role in key developmental processes by regulating the expression of some important genes. Recently, it was observed that animal miRNAs show minimal sequence complementarity with the target sequence; thereby a single miRNA can possibly interact with many genes with similar sequence composition [82]. They are assembled with Argonaute into multiprotein effector complexes, called RNA-induced silencing complexes (RISCs) [83]. MicroRNA can upregulate and downregulate the expression of a gene and in some specific conditions a single gene could encounter both regulation direction [84]. Human miR-373 was the first miRNA to be identified as an activator of gene expression [85]. Corresponding work showed that miRNAs have extensive gene regulatory mechanisms [86- 87]. Similarly, several other researches showed the inhibition / downregulation of gene expression by miRNA through perfect binding with their target genes [88]. MicroRNA mediated regulation of gene expression is selective, specific to the sequence, and depends on the miRNP factors and other RNA binding proteins [89].

## Conclusions:

Transcription, mRNA folding, CpG islands, translation, gene length, GC composition, codon usage bias, amino acid composition and tRNA abundance are essential processes during eukaryotic gene expression, but their relative global contributions to steady-state protein concentrations in multi-cellular eukaryotes are largely unknown. These factors influence gene expression through their interaction with cellular machinery either individually or in combination of these sequence-based factors. Sequence features alone can explain >50% of protein abundance variation. Therefore, while optimizing a gene sequence for heterologous expression a single base pair change can show high degree of co-variation and complex interdependence. Several of these features have hardly been used in synthetic gene design and require more attention in future attempts. Hence, a systematic assessment of all relevant variables is essential to ensure the desired level of protein production. We document factors that need to be examined in details for increasing gene expression in eukaryotes. An understanding of the intricate relationships of the factors in a coordinated approach through establishment of protein expression system is relevant. Targeted study of these constraints in specific disease condition will certainly give novel insights for gene therapies and make significant innovations to ameliorate the specific diseases.
